# Prevention of cisplatin-based chemotherapy-induced delayed nausea and vomiting using triple antiemetic regimens: a mixed treatment comparison

**DOI:** 10.18632/oncotarget.8255

**Published:** 2016-03-22

**Authors:** Qi Shi, Wen Li, Hongjia Li, Qiqi Le, Shanshan Liu, Shaoqi Zong, Leizhen Zheng, Fenggang Hou

**Affiliations:** ^1^ Oncology Department of Municipal Hospital of Traditional Chinese Medicine, Shanghai University of Traditional Chinese Medicine, Shanghai 200071, China; ^2^ Oncology Department of Xin Hua Hospital Affiliated To Shanghai Jiaotong University School of Medicine, Shanghai, 200092, China; ^3^ Digestive Department of Municipal Hospital of Traditional Chinese Medicine, Shanghai University of Traditional Chinese Medicine, Shanghai 200071, China

**Keywords:** chemotherapy-induced nausea and vomiting (CINV), highly emetogenic chemotherapy, cisplatin-based chemotherapy

## Abstract

A variety of triple antiemetic regimens are being used to prevent cisplatin-based chemotherapy induced delayed emesis and nausea in cancer patients. We performed a network meta-analysis to compare the efficacies of the different regimens. Electronic searches of the PubMed, Cochrane Library and MEDLINE databases were performed to identify randomized controlled trials, and data were analyzed using JAGS, Stata 14.0 and R project. The primary outcome was a complete response (CR). The secondary outcomes were no vomiting (NV) and no nausea (NN). Among the 398 studies identified, 10 were eligible and included, providing data on nine regimens. In the CR analysis, the absolute rank of netupitant + palonosetron + dexamethasone (NEPA) was 0.8579. In the NV and NN analyses, NEPA's absolute ranks were 0.8631 and 0.7902, respectively. The compliance of patients treated with rolapitant + granisetron + dexamethasone (RGD) was the best due to a low incidence of adverse events, and good compliance was also observed with NEPA. It was difficult to achieve good compliance with aprepitant + granisetron + dexamethasone (AGD). Overall, NEPA was the best regimen, and aprepitant + ondansetron + dexamethasone (AOD) is also worthy of recommendation because of its low cost and good effect. For patients with severe constipation, hiccups, asthenia and/or delayed nausea, RGD is worthy of consideration.

## INTRODUCTION

Chemotherapy-induced nausea and vomiting (CINV) is a common adverse event in the treatment of cancer and constitutes the main reason for patients’ refusal of chemotherapy [1, 2]. In recent years, although more than 90% of highly emetogenic chemotherapy (HEC)-induced acute vomiting has been effectively controlled using neurokinin-1 (NK-1) and serotonin (5-HT3) antagonists [3–5], 25–35% of delayed vomiting and 60–70% of delayed nausea remain difficult to control [6–9]. The National Comprehensive Cancer Network (NCCN) guidelines for antiemesis (2015.V1) recommend a triple regimen of a NK-1 and 5-HT3 antagonist plus dexamethasone (DXM) to control delayed nausea and vomiting. However, the various regimens in use have never been directly compared, and this lack of information makes it difficult for clinicians to select the optimal antiemetic triple regimen.

Cisplatin, which is widely used in cancer chemotherapy, commonly causes delayed nausea and vomiting [10, 11]. To identify a better triple regimen for cisplatin-based chemotherapy-induced delayed nausea and vomiting, we performed a network meta-analysis of published clinical trials whose outcomes included a complete response (CR), no vomiting (NV), no nausea (NN), and the effects of triple regimens on chemotherapy-related adverse events.

## RESULTS

### Literature search and study characteristics

**Figure 1:**
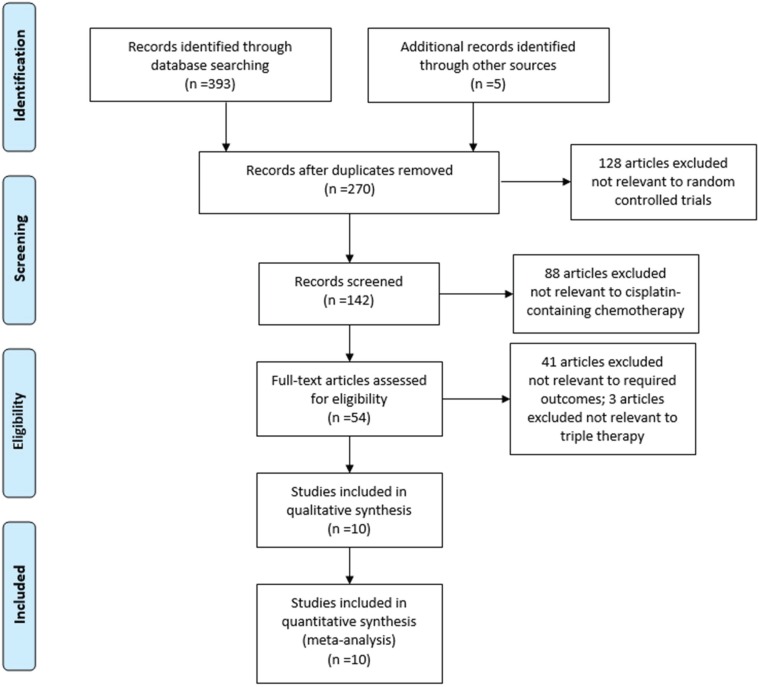
Summary of the identification and selection of clinical trials

A total of 393 citations and 5 additional records were identified in the electronic database search (Figure 1). Of those, 270 potentially relevant articles were retrieved and assessed in greater detail. From that group, 128 studies were excluded because they did not involve randomized controlled trials (RCTs). Also excluded were 88 studies not related to cisplatin-based chemotherapy, 41 that presented uncorrelated outcomes, and 3 that did not include triple therapy. Ultimately, 10 studies [12–21] fulfilled the eligibility criteria (Table 1). The Jadad scores (Supplementary Table S1) of all of the included studies were calculated to be 4–5, indicating that they were of high quality.

**Table 1:** Main characteristics of the studies included in the meta-analysis

Study	Trial design	Patients	Intervention		NV	NN	CR
			Acute phase	Delayed phase			
Paul J. Hesketh, et al. 2003 [12]	parallelgroup double-blind	520	OND 32 mg iv + DXM 20 mg po APR 125 mg po + OND 32 mg iv + DXM 12 mg po	DXM 8 mg po bid APR 80 mg po + DXM 8 mg po on day 2–3, DXM 8 mg on day 4	153/260 (58.9%) 210/260 (80.8%)	124/260 (47.7%) 133/260 (51.2%)	145/260 (55.8%) 196/260 (75.4%)
Sant P. Chawla, et al. 2001 [13]	parallelgroup double-blind	258	APR 125 mg + OND 32 mg iv + DXM 20 mg po Placebo po + OND 32 mg + DXM 20 mg	APR 80 mg + DXM 8 mg Placebo po + DXM 8 mg	102/132 (77.3%) 63/126 (50.0%)	77/132 (58.3%) 46/126 (36.5%)	96/132 (72.7%) 57/126 (45.2%)
Daniel Campos, et al. 2001 [14]	parallelgroup double-blind	174	GRA 10 μg/kg iv + DXM 20 mg po GRA 10 μg/kg + DXM 20 mg po + APR 400 mg po	Placebo po APR 300 mg po	26/90 (28.9%) 53/84 (63.1%)	N/A	N/A
Sergio Poli-Bigelli, et al. 2003 [15]	parallelgroup double-blind	523	OND 32 mg iv + DXM 20 mg po APR 125 mg po + OND 32 mg po + DXM 12 mg po	DXM 8 mg po bid APR 80 mg po + DXM 8 mg po on day 2–3, DXM 8 mg on day 4	126/263 (47.9%) 187/260 (71.9%)	105/263 (39.9%) 138/260 (53.1%)	123/263 (46.8%) 176/260 (67.7%)
P. J. Hesketh, et al. 2014 [16]	parallelgroup double-blind	403	PAL 0.5 mg po + DXM 20 mg po + placebo NETU 300 mg po + PAL 0.5 mg po + DXM 12 mg po APR 125 mg po + OND 32 mg po + DXM 12 mg po APR	DXM 8 mg po bid DXM 4 mg po bid APR 80 mg po + DXM 8 mg po on day 2–3, DXM 8 mg on day 4	109/136 (80.1%) 124/135 (91.9%) 118/132 (89.4%)	110/136 (80.9%) 122/135 (90.4%) 116/132 (87.9%)	109/136 (80.1%) 122/135 (90.4%) 119/132 (90.2%)
H. Saito, et al. 2013 [17]	parallelgroup double-blind	340	FOS 150 mg iv + GRA 40 μg/kg iv + DXM 10 mg iv Placebo iv + GRA 40 μg/kg iv + DXM 20 mg iv	DXM 4 mg iv on day 2, and 8 mg on day 3 DXM 8 mg iv on day 2–3	119/173 (68.8%) 85/167 (50.9%)	53/173 (30.6%) 41/167 (24.6%)	112/173 (64.7%) 81/167 (48.5%)
Toshiaki Takahashi, et al. 2010 [18]	parallelgroup double-blind	295	APR 125 mg po + GRA 40 μg/kg iv + DXM 6 mg iv Placebo po + GRA 40 μg/kg iv + DXM 12 mg iv	APR 80 mg + DXM 4 mg on day 2–3, and APR 80 mg po on day 4–5 Placebo po + DXM 8 mg iv on day 2–3, and placebo po on day 4–5	115/146 (78.8%) 79/149 (53.0%)	51/146 (34.9%) 39/149 (26.2%)	106/146 (72.6%) 77/149 (51.7%)
Zhihuang Hu, et al. 2014 [19]	parallelgroup double-blind	412	APR 125 mg po + GRA 3 mg iv + DXM 6 mg po Placebo po + GRA 3 mg iv + DXM 10.5 mg po	APR 80 mg po + DXM 3.75 mg po on day 2–3, DXM 3.75 mg po on day 4. Placebo po + DXM 7.5 mg po on day 2–3, DXM 7.5 mg po on day 4.	N/A	N/A	151/204 (74.0%) 124/208 (59.6%)
Steven Grunberg, et al. 2011 [20]	parallelgroup double-blind	2322	FOS 150 mg iv + OND 32 mg iv + DXM 12 mg po APR 125 mg po + OND 32 mg iv + DXM 12 mg po	DXM 8 mg po on day 2, 8 mg po bid on day 3–4 APR 80 mg po + DXM 8 mg po on day 3, DXM 8 mg po on day 4	867/1147 (75.6%) 898/1175 (76.4%)	N/A	852/1147 (74.3%) 872/1175 (74.2%)
Bernardo L Rapoport, et al. 2015 [21]	parallelgroup double-blind	1070	ROL 180 mg po + GRA 10 μg/kg iv + DXM 20 mg po GRA 10 μg/kg iv + DXM 20 mg po	DXM 8 mg po bid DXM 8 mg po bid	404/535 (75.6%) 340/535 (63.6%)	298/535 (55.7%) 237/535 (49.9%)	382/535 (71.4%) 322/535 (60.2%)

Abbreviations: N/A, no adequate data in relevant trials.

FOS, Fosaprepitant; APR, aprepitant; PAL, palonosetron; OND, ondansetron; GRA, granisetron: DXM, dexamethasone; NETU, netupitant; ROL, rolapitant;

CR, complete response; NN, no nausea; NV, no vomiting.

### Risk of bias

The methodological quality of the included studies was generally good. Across all six domains, approximately 71.6% of the assessments were classified as ‘low risk’, and 1.7% were classified as ‘high risk’. It is unlikely that the evidence presented in this review was affected by biases associated with performance. However, we cannot exclude the possibility that selection bias was present in some individual trials, since they lacked a description of their allocation. In addition, some uncertainty regarding the risks of bias associated with random sequence generation and with the blinding of outcome assessors was due mainly to insufficient reporting. The risk of bias in the included studies is summarized in Supplementary Figures S1–S2.

### Traditional meta-analysis

**Figure 2:**
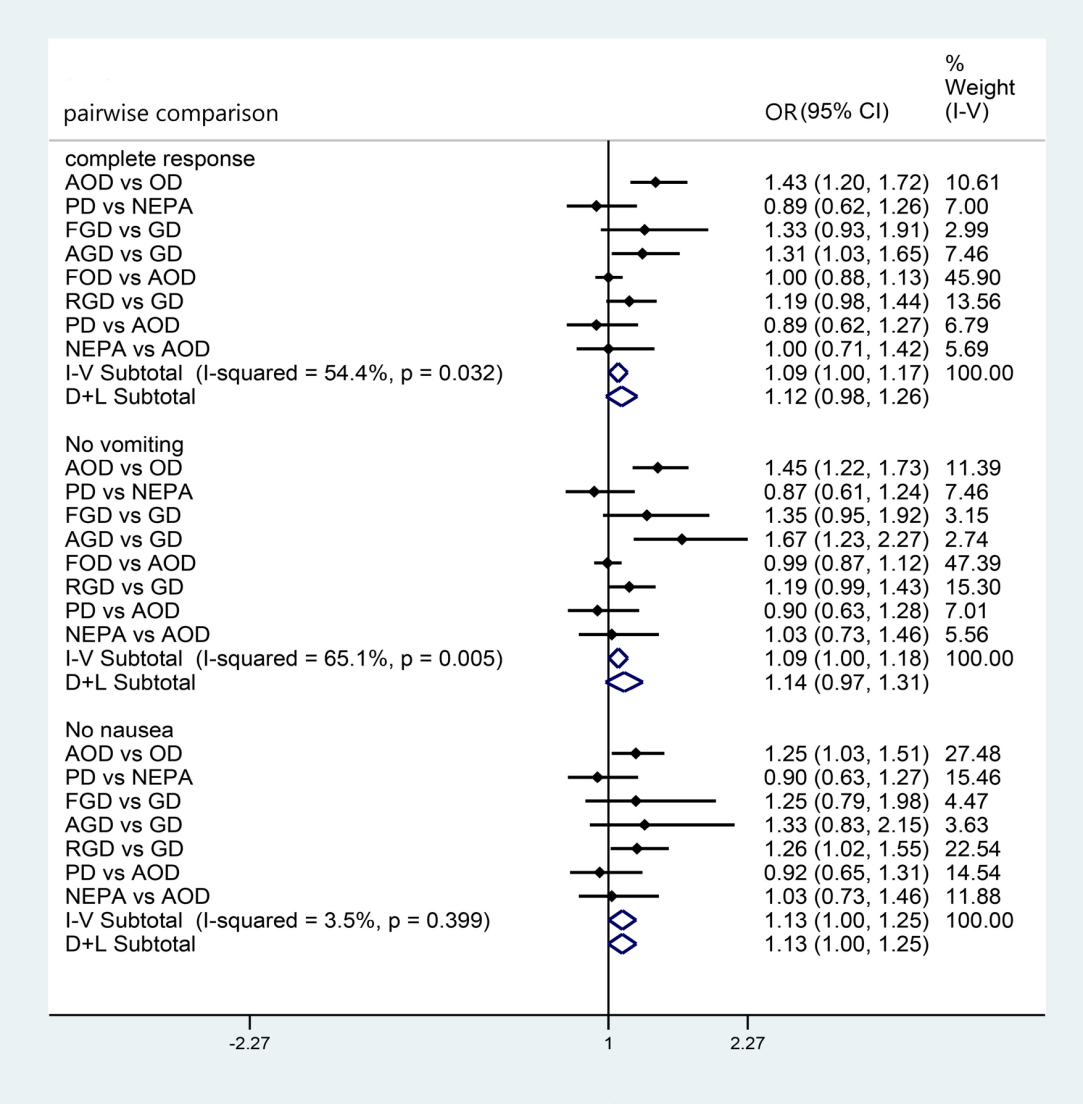
Meta-analysis of pairwise comparisons for effects on primary and secondary outcome Direct comparison of included trials were analysed using a random effect model. Odd ratios and confidence intervals are shown on the right side of the table. I^2^ and *P* values indicate the heterogeneity in each outcome.

We performed a series of pairwise meta-analyses to evaluate antiemetic regimens. Figure 2 shows that most of the comparisons did not reveal significant differences for CR and NV, though the ORs were significantly better with aprepitant + ondansetron + dexamethasone (AOD) than with ondansetron + dexamethasone (OD), and were better with aprepitant + granisetron + dexamethasone (AGD) than with granisetron + dexamethasone (GD). In addition, we found that the *I*^2^ values were > 50% for analyses of CR and NV, indicating acceptable levels of heterogeneity. For analysis of NN, however, the ORs were significantly better for AOD than for OD and for rolapitant + ondansetron + dexamethasone (RGD) than for GD. In addition, no significant heterogeneity was detected, with an *I*^2^ value of > 50%.

Because heterogeneity was detected in the CR and NV analyses, we conducted sensitivity analyses to verify the stability of the results. As shown in Figure 2, the small-sample study had no substantial impact on the results. However, the study by Grunberg et al. [20] had the greatest effect on the combined results (Supplementary Figure S3). We suggest the heterogeneity detected may have been due to the significantly larger sample size in this study than in the other studies.

### Network meta-analysis (combination of direct and indirect comparisons)

We used a fixed effects model to analyze the data because it provides a narrower interval estimation. Table 2 shows an evaluation of consistency in CR. Each partition node shows the differences between the direct and indirect results. The corresponding *P* values are > 0.05; there is thus no evidence that the network model is inconsistent.

**Table 2:** Evaluation of consistency for primary outcome (complete response)

Side	Direct		Indirect		Difference		*P*
	Coef	Std. Err.	Coef	Std. Err.	Coef.	Std. Err.	
A E	.7639229	.1615678	.6257291	130.9475	.1381938	130.9476	0.999
B G	.935809	.1176838	.1210314	57.23982	.8147776	57.23995	0.989
B D	.0035546	.0949245	1.873384	145.1808	1.876939	145.1808	0.990
B F	.0248976	.4128513	1.194039	187.4053	1.218937	187.4058	0.992
C H	.8186631	.3626814	2.037544	187.729	1.218881	187.7295	0.995
C E	.6675231	.2220261	.64144	148.881	.0260831	148.8811	1.000
E I	.5017233	.1302082	.7175594	153.7626	.2158361	153.7627	0.999

This result is based on a node-splitting model. Direct estimates are always compared with indirect ones. If the *P* value is > 0.05, the comparison for this node is not inconsistent.

Abbreviations: Coef, regression coefficient; Std. Err., Standard error.

**Figure 3:**
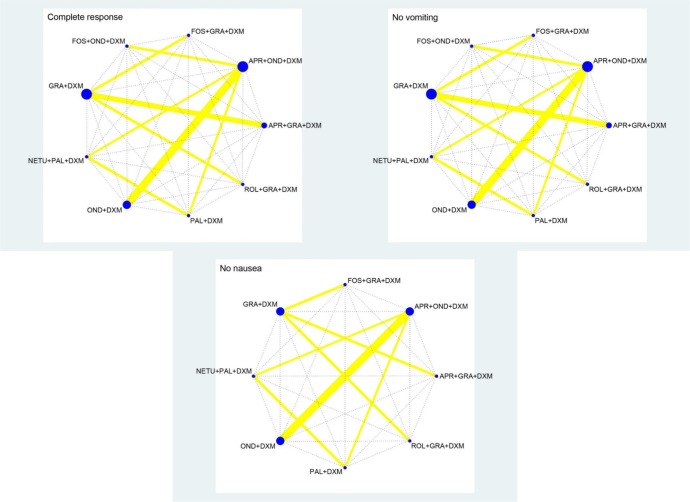
Network structures for all outcomes Solid lines link treatments directly compared in trials, and dotted lines indicate the lack of a direct comparison between treatments. The thicknesses of the solid lines are proportional to the numbers of comparisons included in the network. The diameters of the circles are proportional to the numbers of studies involving specific treatments. Abbreviations: NEPA, NETU + PAL + DXM; AOD, APR + OND + DXM; FOD, FOS + OND + DXM; AGD, APR + GRA + DXM; PD, PAL + DXM; FGD, FOS + GRA + DXM; RGD = ROL + GRA + DXM; OD, OND + DXM; GD, GRA + DXM.

Figure 3 shows the network structures for CR, NV and NN. Each solid line links treatments directly compared within a trial, while each dotted line indicates a lack of direct comparison between treatments. The thicknesses of the solid lines are proportional to the number of comparisons included in the network, and the diameters of the circles are proportional to the number of studies involving the specific treatments.

### Efficacy endpoint

#### Complete response (CR)

**Figure 4:**
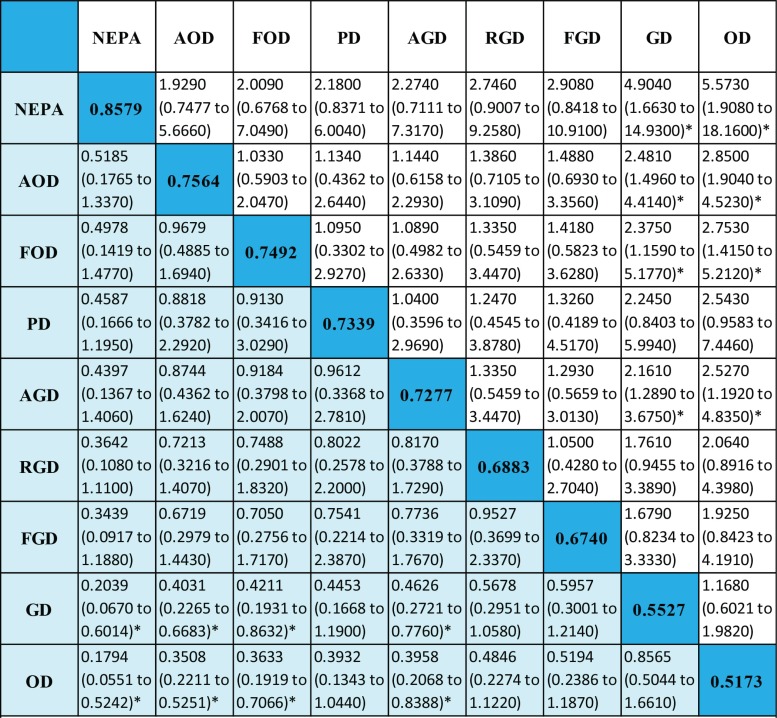
Efficacy of antiemetic regimens for a complete response Ranges in parentheses are 95% CIs. An OR more than 1 shows that the regimen listed in the left column is more beneficial than the one in the top row. Regimens are ordered according to their efficacy ranking. Absolute ranks are given in the diagonal. The larger the absolute rank, the better the treatment. Abbreviations, OD, ondansetron + dexamethasone; GD, granisetron + dexamethasone; PD, palanosetron + dexamethasone; AOD, aprepitant + ondansetron + dexamethasone; FOD, fosaprepitant + ondansetron + dexamethasone; AGD, aprepitant + granisetron + dexamethasone; FGD, fosaprepitant + granisetron + dexamethasone; NEPA, netupitant + palonosetron + dexamethasone; RGD, rolapitant + granisetron + dexamethasone.

Figure 4 shows the preventive effect of 8 antiemetic regimens on delayed vomiting, with the outcomes of a total of 6,143 patients being reported. CR analysis revealed that netupitant + palonosetron + dexamethasone (NEPA) was the most effective treatment, with an absolute rank of 0.8579 The ranking from high to low was as follows: AOD, fosaprepitant + ondansetron + dexamethasone (FOD), palonosetron + dexamethasone (PD), AGD, RGD, fosaprepitant + granisetron + dexamethasone (FGD), GD and OD. However, the results should be interpreted with caution because most of comparisons among the various regimens did not reach statistical significance.

### No vomiting (NV)

**Figure 5:**
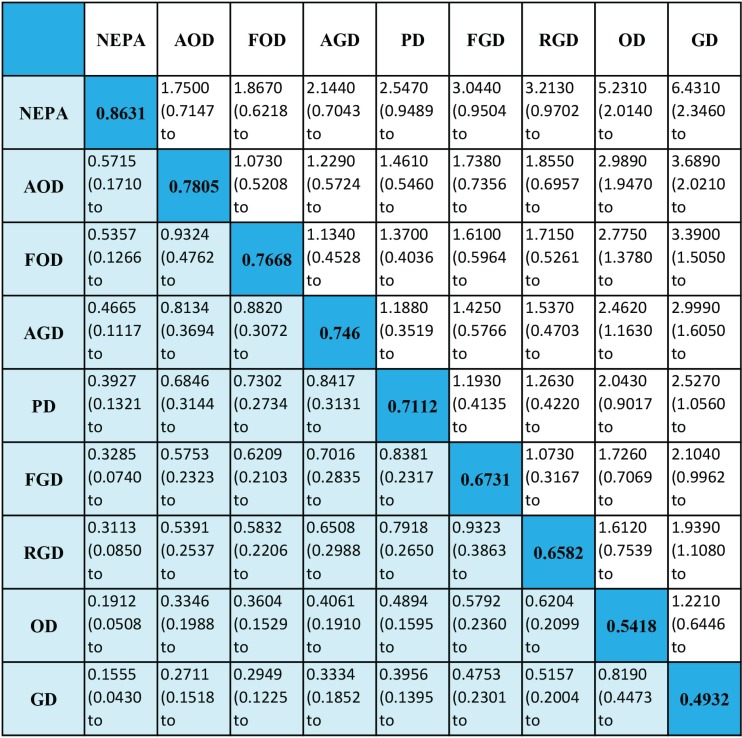
Efficacy of antiemetic regimens for no vomiting Ranges in parentheses are 95% CIs. An OR more than 1 shows that the regimen listed in the left column is more beneficial than the one in the top row. Regimens are ordered according to their efficacy ranking. Absolute ranks are given in the diagonal. The larger the absolutely rank, the better the treatment. Abbreviations: OD, ondansetron + dexamethasone; GD, granisetron + dexamethasone; PD, palanosetron + dexamethasone; AOD, aprepitant + ondansetron + dexamethasone; FOD, fosaprepitant + ondansetron + dexamethasone; AGD, aprepitant + granisetron + dexamethasone; FGD, fosaprepitant + granisetron + dexamethasone; NEPA, netupitant + palonosetron + dexamethasone; RGD, rolapitant + granisetron + dexamethasone.

The results of the NV analysis are shown in Figure 5. In nine studies, a total of nine antiemetic regimens and 4,835 patients were analyzed. The absolute rank of NEPA was 0.8631, which indicates this regimen may be optimal. The emetic regimens in decreasing order of absolute rank were as follows: AOD, FOD, AGD, PD, FGD, RGD, OD and GD. Again, these findings should be interpreted with caution because most of the comparisons did not reach statistical significance.

### No nausea (NN)

**Figure 6:**
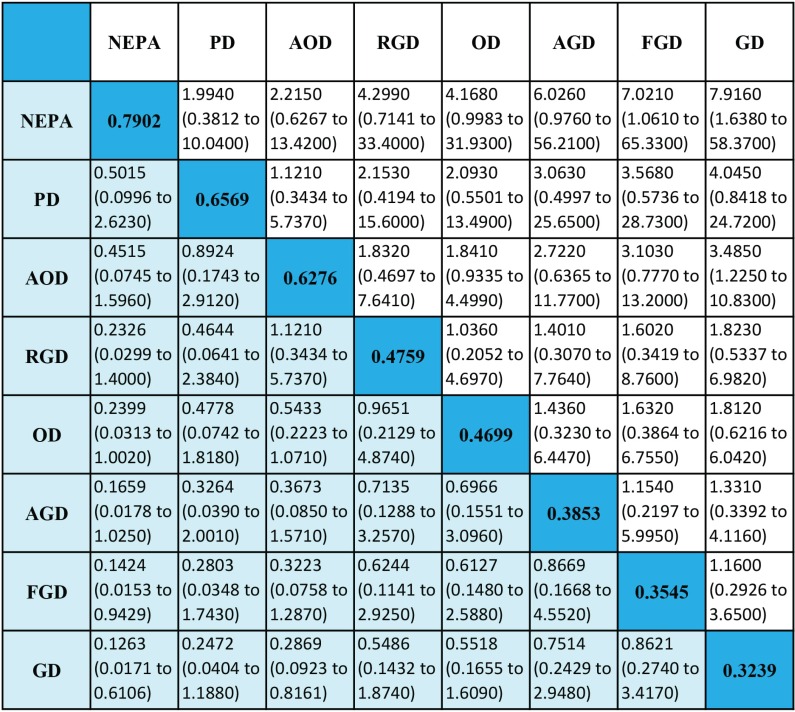
Efficacy of antiemetic regimens for no nausea Ranges in parentheses are 95%CIs. An OR more than 1 shows that the regimen listed in the left column is more beneficial than the one in the top row. Regimens are ordered according to their efficacy ranking. Absolute ranks are given in the diagonal. The larger the absolute rank, the better the treatment. Abbreviations: OD, ondansetron + dexamethasone; GD, granisetron + dexamethasone; PD, palanosetron + dexamethasone; AOD, aprepitant + ondansetron + dexamethasone; AGD, aprepitant + granisetron + dexamethasone; FGD, fosaprepitant + granisetron + dexamethasone; NEPA, netupitant + palonosetron + dexamethasone; RGD, rolapitant + granisetron + dexamethasone.

The results of the NN analysis are shown in Figure 6. In seven studies, a total of eight antiemetic regimens and 3,409 patients were analyzed. The FOD regimen was not analyzed in this section because the studies in which it was included did not report the relevant data. The efficacy of NEPA was again the best, with an absolute rank of 0.7902. In decreasing order, the ranking was as follows: PD, AOD, RGD, OD, AGD, FGD, and GD. These results should also be interpreted with caution.

### Safety

The incidence of adverse events among the patients receiving the different triple antiemetic regimens are shown in Table 3. The incidences of constipation (23.9%), anorexia (36.3%) and hiccups (35.5%) were the highest in the patients treated with AGD, while the incidence of asthenia (14.3%) was the highest in the patients treated with AOD. The incidence of adverse events (constipation: 0.4%, hiccups: 0.6% and asthenia: 0.4%) was the lowest in patients treated with RGD.

**Table 3:** Incidence of adverse events in patients treated with different triple antiemetic regimens

Regimen	Constipation	%	Hiccups	%	Asthenia	%	Anorexia	%	Diarrhea	%
OD	95/761	12.5	37/476	7.8	101/761	13.3	62/497	12.5	55/497	11.1
AOD	198/1926	10.3	136/1778	7.6	276/1926	14.3	175/1799	9.7	160/1665	9.6
GD	105/948	11.1	127/1158	11	40/837	4.8	100/241	41.5	61/451	13.5
FGD	23/174	13.2	15/174	8.6	N/A	N/A	N/A	N/A	N/A	N/A
AGD	105/439	23.9	83/234	35.5	31/289	10.7	85/234	36.3	56/445	12.6
PD	N/A	N/A	5/136	3.7	N/A	N/A	3/136	2.2	N/A	N/A
FOD	121/1143	10.6	64/1143	5.6	98/1143	8.6	76/1143	6.7	89/1143	7.8
NEPA	N/A	N/A	7/136	5.1	N/A	N/A	1/136	0.7	N/A	N/A
RGD	2/535	0.4	3/535	0.6	2/535	0.4	N/A	N/A	N/A	N/A
Total	649/5926	11.0%	540/5770	9.4%	548/5491	10%	502/4186	12.0%	421/4201	10.0%

Shown are the incidences of adverse events in patients treated with the indicated regimens. Total incidences of constipation, hiccups, asthenia, anorexia and diarrhea are shown at the bottom of the table. N/A, no adequate data in relevant trials.

NEPA, NETU + PAL + DXM; AOD, APR + OND + DXM; FOD, FOS + OND + DXM; AGD, APR + GRA + DXM; PD, PAL + DXM; FGD, FOS + GRA + DXM; RGD = ROL + GRA + DXM; OD, OND + DXM; GD, GRA + DXM.

## DISCUSSION

In recent years, the prevention of CINV has been greatly improved by the widespread utilization of 5-HT3 and NK-1 antagonists. Although drugs in the same categories are unlikely to have different antiemetic properties, studies of triple regimens aimed at treating delayed nausea and vomiting have nonetheless received significant attention. The current evidence indicates that the efficacy of triple regimens is generally better than that of double regimens because of the interaction between NK-1 and 5-HT3 receptor antagonists [22–27]. However, the differences among triple antiemetic regimens have not yet been directly compared. Therefore, we evaluated several commonly used triple regimens with regard to CR, NV, NN and safety.

### Complete response

The results of our CR analysis revealed that NEPA may be the most effective regimen (absolute rank: 0.8579), though the efficacies of AOD and FOD ranked second and third (absolute rank 0.7564 and 0.7492, respectively) and did not significantly differ from NEPA. Thus all three of these regimens generate a good CR. Notably, the absolute rank of PD was 0.7339, and it exhibited a tendency to be more effective than AGD and RGD.

### Delayed vomiting

Many studies have shown that NK-1 antagonists enhance the efficacy of 5-HT3 antagonists through induction or inhibition of substance *P* [28–31]. However, these two types of drugs can be combined to form various triple regimens, and it is not yet clear whether the different combinations have different abilities to prevent delayed vomiting. NV analysis revealed that NEPA may be the most effective regimen (absolute rank: 0.8631) and that AOD and FOD are ranked second and third (absolute ranks: 0.7805 and 0.7668, respectively). Furthermore, there were no obvious differences among the triple regimens evaluated, indicating that they do not significantly differ in their abilities to prevent delayed vomiting. Thus, among the regimens examined, the efficacy of NEPA appeared to be the best, but the other emetic regimens also produced good effects.

### Delayed nausea

Delayed nausea has gradually become the focus of CINV research. The ability of an antiemetic regimen to preventing nausea may differ its ability to prevent vomiting because different mechanisms are involved [32]. NN analysis revealed that RGD ranked forth (absolute rank: 0.4759), whereas this regimen ranked seventh in NV analysis; thus RGD appears to have better effects against delayed nausea than delayed vomiting. In addition, NEPA was ranked first (absolute rank: 0.7902) in NN analysis, which indicates this regimen may be the most effective for preventing delayed nausea. However, no remarkable differences were detected among the triple regimens in the NN analysis, which suggests all of these regimens are similarly preventative against delayed nausea.

### Safety

Because treatment-related adverse events often affect patients’ tolerances, they are always included in evaluations of the safety of antiemetic regimens. Among these events, constipation, hiccups, asthenia, anorexia and diarrhea were the most commonly reported in previous studies [33]. We therefore focused on these five adverse events in our analyses.

With regard to constipation, the RGD and AGD regimens were associated with the lowest and highest incidences, respectively (0.4% and 23.9%, respectively). The other triple regimens exhibited relatively small differences in the incidence of constipation. Gralla et al. and Aapro et al. [34, 35] reported that the incidences of constipation in patients treated with NEPA are 3.6% and 2.1%, respectively, which are similar to the value obtained for the RGD regimen in this study. With regard to hiccups, the RGD and AGD regimens were associated with the lowest and highest incidences, respectively (0.6% and 35.5%, respectively). The other triple regimens exhibited relatively small differences in their incidences of hiccups. The RGD regimen was also associated with the lowest incidence asthenia (0.4%). No information regarding the incidence of asthenia associated with NEPA was available from the included studies; however, Calcagnile et al. and Lanzarotti et al. [36, 37] reported incidences of 10% and 13.9%, respectively, which are similar to the other triple regimens in this study. The NEPA and AGD regimens were respectively associated with the lowest and highest incidences of anorexia (0.7% and 36.3%, respectively). The incidences of anorexia did not significantly differ among the other triple regimens. Finally, the incidences of diarrhea among all of the triple regimens ranged from 7.8% to 12.6%, and the differences among them were not significant.

Overall, based on the incidences of adverse events, we hypothesize that the best compliance rate would be achieved by treating patients with RGD. Previous reports indicate that rolapitant differs from other NK-1 antagonists [37–40] in that it is not metabolized by CYP P450 3A4 (CYP3A4) [41, 42]. Consequently, this drug likely avoids drug-drug interactions and potential adverse events [43, 44]. Patients treated with NEPA also showed good compliance. By contrast, among all of the triple regimens evaluated, patients treated with AGD had the most difficulty achieving good compliance.

### Limitations

Previous studies have shown that patients with osteosarcoma or soft tissue sarcoma do not respond to NK-1 + 5-HT3 + DXM regimens, suggesting the efficacies of triple regimens for preventing CINV may be related to the cancer type [45, 46]. We could not perform subgroup analysis of specific cancer types because data on cancer types were lacking in the included studies. Thus, we could not clearly determine whether the efficacies of the triple regimens differed based on the cancer type.

## MATERIALS AND METHODS

### Search

This meta-analysis was conducted in accordance with the Preferred Reporting Items for Systematic Reviews and Meta-Analyses (PRISMA) guidelines [47, 48]. A comprehensive literature search of the PubMed, MEDLINE, EMBASE, and the Cochrane Library databases was performed. We used the terms “cisplatin”, “CINV” or “chemotherapy induced nausea and vomiting” in combination with “highly emetogenic chemotherapy” and “randomized controlled clinical trials” to identify studies related to CINV. Additionally, we reviewed the reference lists of all meta-analyses and other publications as potential data sources. When data or study characteristics were not reported in the primary publication, we searched clinical trial reports, trial registries and drug company websites to obtain additional data. When possible, we used data from intention-to-treat (ITT) analyses for all randomly assigned participants.

### Inclusion criteria

The eligibility criteria included enrollment of patients receiving cisplatin-containing chemotherapy. Trials were excluded if: 1) they were not randomized; 2) the intervention was not relevant to cisplatin-based chemotherapy; 3) no triple regimen was assessed; 4) they were published in a language other than English; or 5) the trial results were not relevant to delayed nausea and vomiting. Two independent reviewers screened all of the retrieved references based on these predefined exclusion criteria. A two-round process was used; titles and abstracts were screened for potential relevance prior to reviewing full text publications.

### Data extraction

Two researchers independently extracted the following data from each eligible study: the first author, year of publication, trial design, intervention, outcome indicator, and numbers of cases and controls. To ensure accuracy of the data, inconsistencies were discussed by the researchers so as to reach a consensus.

### Risk of bias assessment

We assessed the included studies using The Cochrane Collaboration's “Risk of bias (RoB)” tool outlined in Table 8.5c of the Cochrane Handbook for Systematic Reviews of Interventions, after which the assessment was checked by a second review author. We considered adequate sequence generation and allocation concealment to be most important in this assessment; therefore, a judgment of low risk was desirable for these domains for all trials. Blinding was not appropriate due to the nature of the treatments, and any issues regarding the reporting of incomplete outcome data, selective outcome reporting, or attrition bias were overcome by the collection of individual studies.

### Quality assessment

We assessed the quality of each study according to quality assessment criteria (Jadad scale). The quality scores of the studies ranged from 0 (lowest) to 5 (highest). Studies with scores of less than 2 were considered low quality, and those with scores equal to or greater than 3 were regarded as high quality.

### Data analysis

We performed traditional pairwise meta-analysis for direct treatment comparisons. As all of the results were extracted as binary outcomes, we calculated the summary effect sizes as odds ratios (ORs) with 95% confidence intervals. The statistical heterogeneity among studies was assessed using Cochran's *Q* test and the *I*^2^ statistic [49]. A *P* value of 0.10 or less for the *Q* test or an *I*^2^ value of greater than 50% was suggestive of substantial between-study heterogeneity. If heterogeneity was detected, we performed a sensitivity analysis to explore the potential sources of the heterogeneity.

We analyzed the pooled data for all antiemetic regimens using a fixed effects model within a Bayesian framework with the pcnetmeta package of R project [50]. All models were run with 1000 burn-in iterations and at least 50,000 inference iterations [51]. Summary effect sizes were calculated as ORs with 95% creditable intervals [50]. To assess the efficacies of the different regimens, we also calculated their absolute ranks. The resultant rankings are presented graphically.

We also analyzed inconsistencies between the direct and indirect estimates for the primary outcome. Differences between these estimates were detected using a node-splitting model, which used different parameters to divide the comparisons. The model then jointly estimated the two parameters and reported the difference. Finally, the model tested whether the real difference was zero [52].

For traditional meta-analyses we used Stata 14.0. For network meta-analyses we used JAGS, Stata 14.0 and R project.

## CONCLUSIONS

With respect to CR, NV and NN, NEPA had the best preventive effect against cisplatin-based chemotherapy-induced delayed nausea and vomiting. The safety of NEPA was also better, making it worthy of recommendation. AOD ranked second, second and third, respectively, for CR, NV and NN, which also indicates superior preventative effects. From an economic perspective, although the safety of AOD has not been shown to be advantageous, this regimen is also worthy of recommendation because of its low cost. And although RGD offers no advantage with respect to delayed vomiting, it effectively prevents delayed nausea and is relatively safe to use. Thus, the RGD regimen deserves the attention of clinicians and patients for its ability to prevent severe constipation, hiccups, asthenia and/or delayed nausea.

## SUPPLEMENTARY MATERIALS FIGURES AND TABLES


