# Supplementary material for: Prevention of cisplatin-based chemotherapy-induced delayed nausea and vomiting using triple antiemetic regimens: a mixed treatment comparison
Source: Oncotarget. 2016 Mar 22;7(17):24402–14. doi: 10.18632/oncotarget.8255 (PMC5029710; doi:10.18632/oncotarget.8255)
Supplement: Supplementary file 1 [file oncotarget-07-24402-s001.pdf]

Prevention of cisplatin-based chemotherapy-induced delayed nausea and vomiting using triple antiemetic regimens: a mixed treatment comparison

Supplementary Materials

Supplementary Table S1: Jadad quality scale

Study	Random sequence generation	Double blind	Incomplete outcome data	Total score
Daniel Campos 2001	2	2	1	5
H. Saito 2012	1	2	1	4
P. J. Hesketh 2014	1	2	1	4
P. J. Hesketh 2003	2	2	1	5
Sant P. Chawla 2001	2	2	1	5
Sergio Poli-Bigelli 2003	2	2	0	4
Steven Grunberg 2011	2	2	0	4
Toshiaki Takahashi 2010	1	2	1	4
Zhihuang Hu 2014	2	2	1	5
Bernardo Rapoport 2015	2	2	1	5

Jadad quality scale: 1–2 total score is for low quality, 3–5 total score is for high quality.

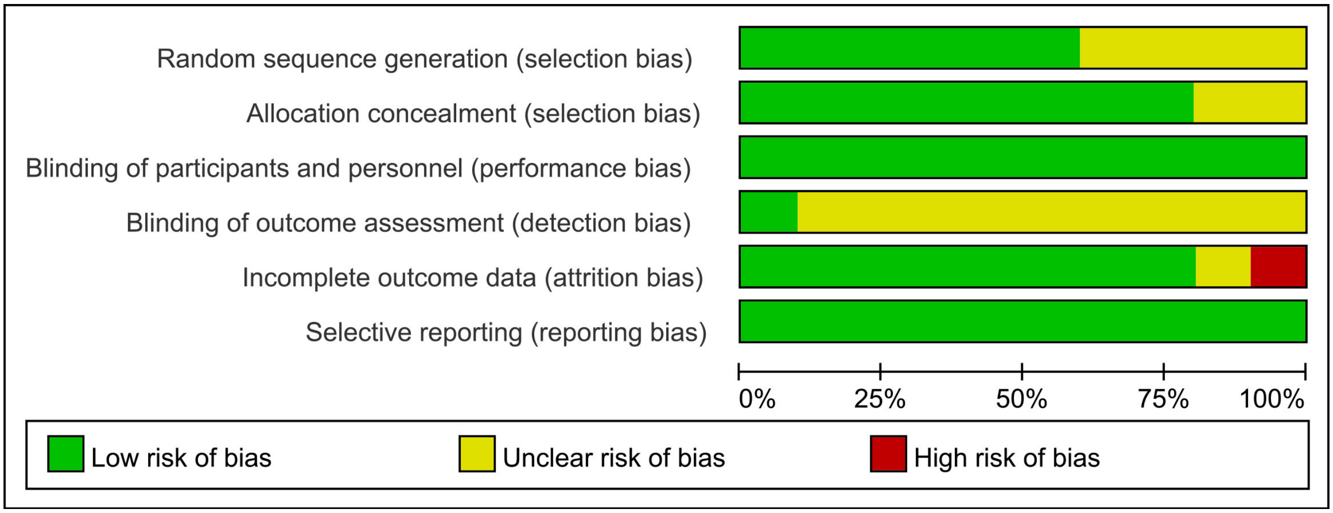

Supplementary Figure S1: Risk of bias graph: review authors' assessment of each risk of bias item presented as percentages across all included studies.

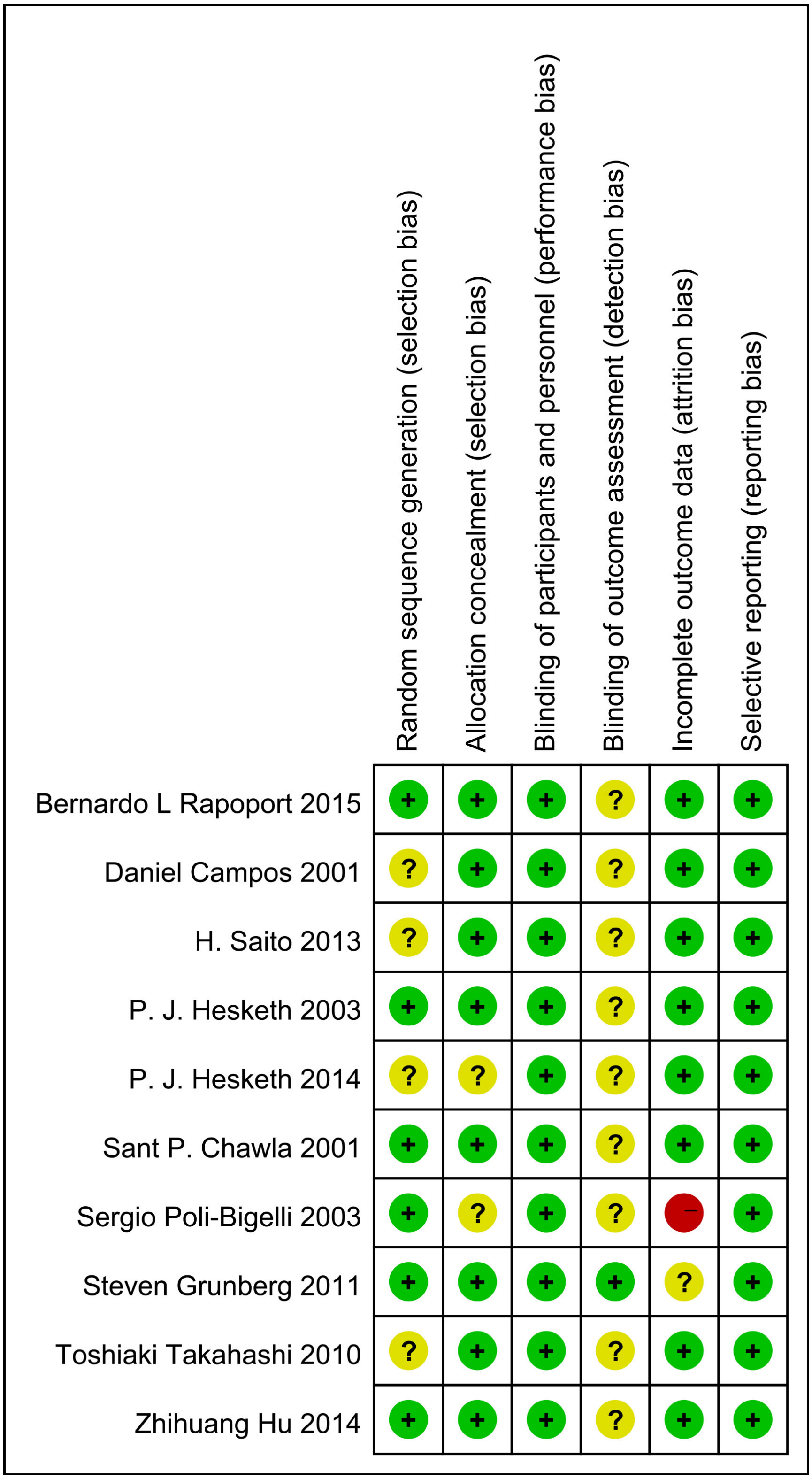

Supplementary Figure S2: Risk of bias summary: review authors' assessment of each risk of bias item for each included study.

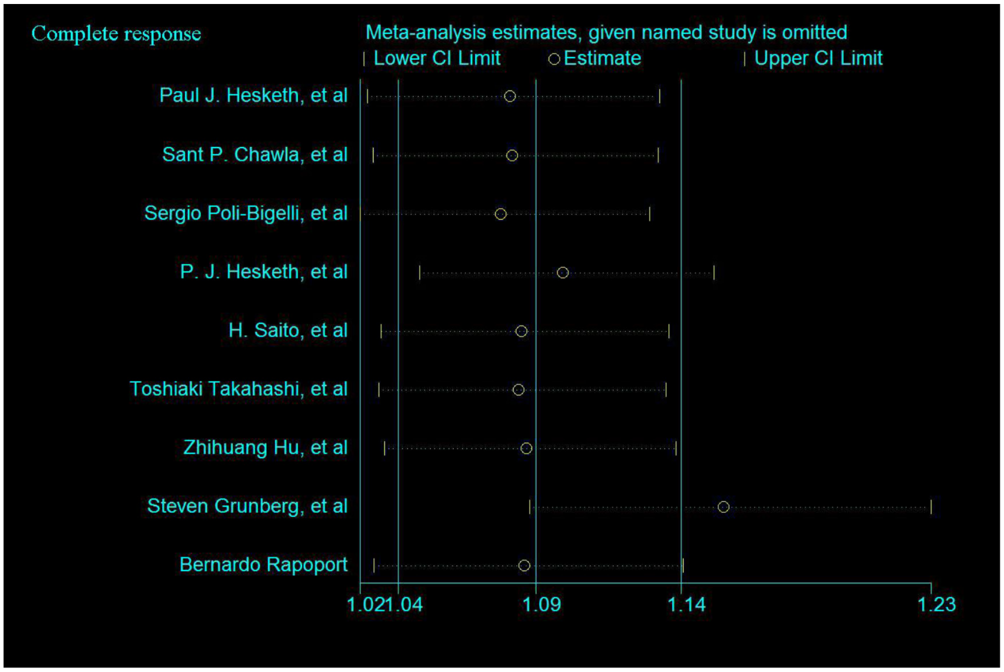

The vertical line at 1.09 represents the total combined effect of a complete response. The two vertical lines on the left and right represent the corresponding upper and lower confidence interval (CI) limits for the total combined effect. The lines corresponding to each study represent the bias caused by the single study.

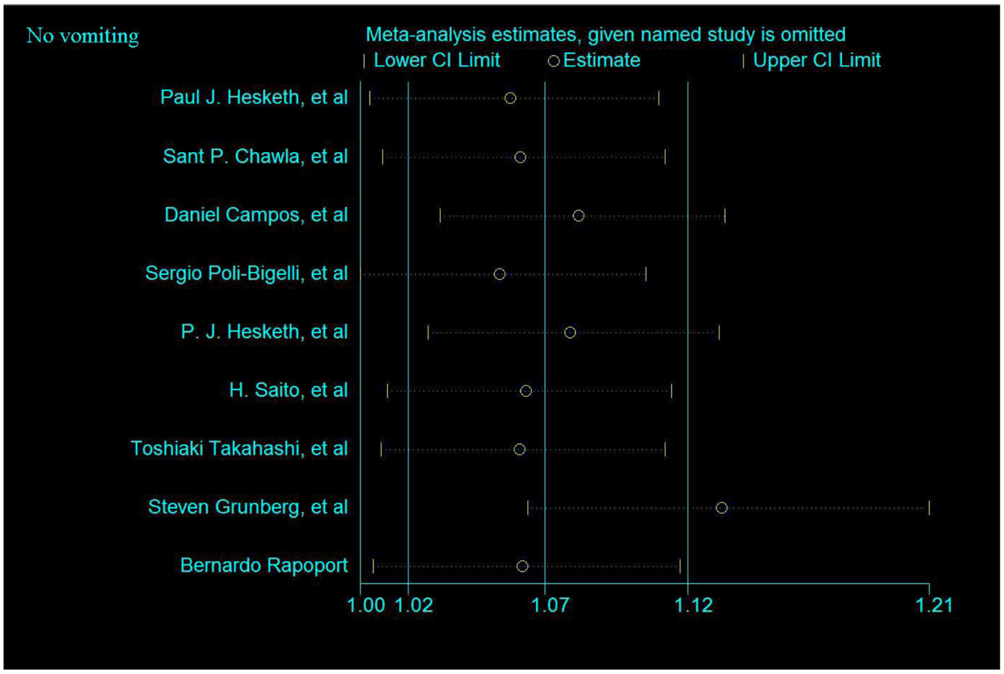

The vertical line at 1.07 represents the total combined effect of no vomiting. The two vertical lines on the left and right represent the corresponding upper and lower confidence interval (CI) limits of the total combined effect. The lines corresponding to each study represent the bias caused by the single study.

Supplementary Figure S3: Sensitivity analysis on complete response (CR) and no vomiting (NV).

Supplementary Table S2: Frequency of adverse events in the included studies

	Constipation	Hiccups	Asthenia	Anorexia	Diarrhea
Daniel Campos	1	1	1	1	1
H. Saito	1	1	0	0	0
P. J. Hesketh 2014	0	1	0	1	0
P. J. Hesketh 2003	1	1	1	0	0
Sant P. Chawla	1	1	1	1	1
Sergio Poli Bigelli	1	0	1	1	1
Steven Grunberg	1	1	1	1	1
Toshiaki Takahashi	1	1	0	1	1
Zhihuang Hu	1	0	1	0	1
Bernardo Rapoport	1	1	1	0	0
Total	9	8	7	6	6

1, the specific adverse event was reported in the study; 0, the specific adverse event was not reported in the study.
